# Chronic upregulation of activated microglia immunoreactive for galectin-3/Mac-2 and nerve growth factor following diffuse axonal injury

**DOI:** 10.1186/1742-2094-7-32

**Published:** 2010-05-27

**Authors:** Charu Venkatesan, MaryAnn Chrzaszcz, Nicole Choi, Mark S Wainwright

**Affiliations:** 1 Division of Neurology, Department of Pediatrics, Northwestern University Feinberg School of Medicine, Chicago, IL, 60614, USA; 2 Center for Interdisciplinary Research in Pediatric Critical Illness and Injury, Northwestern University Feinberg School of Medicine, Chicago, IL, 60614, USA

## Abstract

**Background:**

Diffuse axonal injury in patients with traumatic brain injury (TBI) can be associated with morbidity ranging from cognitive difficulties to coma. Magnetic resonance imaging scans now allow early detection of axonal injury following TBI, and have linked cognitive disability in these patients to white matter signal changes. However, little is known about the pathophysiology of this white matter injury, and the role of microglial activation in this process. It is increasingly recognized that microglia constitute a heterogeneous population with diverse roles following injury. In the present studies, we tested the hypothesis that following diffuse axonal injury involving the corpus callosum, there is upregulation of a subpopulation of microglia that express the lectin galectin-3/Mac-2 and are involved in myelin phagocytosis.

**Methods:**

Adult mice were subject to midline closed skull injury or sham operation and were sacrificed 1, 8, 14 or 28 days later. Immunohistochemistry and immunofluorescence techniques were used to analyze patterns of labelling within the corpus callosum qualitatively and quantitatively.

**Results:**

Activated microglia immunoreactive for galectin-3/Mac-2 were most abundant 1 day following injury. Their levels were attenuated at later time points after TBI but still were significantly elevated compared to sham animals. Furthermore, the majority of galectin-3/Mac-2+ microglia were immunoreactive for nerve growth factor in both sham and injured animals.

**Conclusions:**

Our results suggest that galectin-3/Mac-2+ microglia play an important role in the pathogenesis of diffuse axonal injury both acutely and chronically and that they mediate their effects, at least in part by releasing nerve growth factor.

## Background

Traumatic brain injury (TBI) is a leading cause of mortality and morbidity in Western industrialized nations and poses significant financial and medical burden to society [1]. Neurologic morbidity among survivors is high and includes cognitive impairment, dementia, epilepsy and depression [2-4]. Magnetic resonance imaging (MRI) of survivors with neurocognitive deficits show significant injury to the subcortical white matter, suggesting that damage to these tracts may contribute to deficits in cortical information processing observed in humans [5]. Pathological changes have been observed using diffusion tensor MRI even in patients with mild TBI who have no evidence of overt hemorrhage within the white matter [6]. However, little is known about the role of microglia in the pathophysiology of white matter injury following TBI.

The dual role of microglia following injury is now well-recognized. For example, within the spinal cord, two functionally distinct populations of macrophages have been identified: M1 macrophages that express markers including iNOS, CD86 and MHCII and have a neurotoxic function and M2 macrophages that express Arginase I and CD206 and have a neurotrophic role [7]. Following spinal cord injury, chronic upregulation in M1 macrophages is seen while the increase in M2 macrophages is brief, lasting at most 7 days [7]. In the injured brain, activated microglia have been shown to secrete pro-inflammatory cytokines such as interleukin (IL)-1β, tumor necrosis factor (TNF) α, and IL-6 [8-11] and are involved in phagocytosis of axonal and neuronal debris [12-14]. They have also been shown to have a neuroprotective role following neurological insult via secretion of trophic growth factors such as brain-derived neurotrophic factor, glial cell line-derived neurotrophic factor and insulin-like growth factor [15-18].

Previous studies have shown that a specific subset of activated microglia express the lectin galectin-3/Mac-2, a member of the galectin family of β-galactoside binding lectins [19-21]. Within the central and peripheral nervous system, galectin-3/Mac-2 is expressed by microglia, macrophages and Schwann cells that phagocytose myelin following induction of experimental allergic encephalomyelitis, ischemia, and sciatic nerve transection [20,22,23]. Galectin-3/Mac-2 is not expressed by these cells in the intact nervous system and has not been found within microglia in areas of inefficient myelin phagocytosis following injury [20,22,23]. Taken together, these studies suggest that galectin-3/Mac-2 serves as a marker of a subpopulation of activated microglia involved in myelin degradation.

There is emerging evidence that galectin-3/Mac-2-immunoreactive (ir) microglia may have a role beyond myelin phagocytosis. Following ischemic injury, galectin-3/Mac-2 expressing microglia have been shown to express trophic factors such as insulin-like growth factor in the gray matter [16]. Selective ablation of galectin-3/Mac-2 positive microglia results in an increase in the size of the infarct zone, suggesting a neuroprotective effect of this subset of microglia following ischemic injury [16].

In these studies, we explored whether there is up regulation of galectin-3/Mac-2 immunoreactive microglia within the corpus callosum following diffuse axonal injury and whether these microglia are a potential source of another trophic factor, nerve growth factor (NGF), in the injured subcortical white matter. We specifically chose to evaluate NGF because recent studies of human TBI patients have found an elevation in NGF within the cerebrospinal fluid following injury and have noted a correlation between increased NGF levels and improved clinical outcome [24,25]. We report here that after TBI, there is chronic upregulation of galectin-3/Mac-2 immunoreactive microglia in the corpus callosum that persists at 28 days following injury. Furthermore, we show that almost all galectin-3/Mac-2 immunoreactive microglia are immunoreactive for NGF protein in uninjured and injured animals at all time points examined. Our findings suggest that this subtype of activated microglia plays an important role in the acute and chronic phases of recovery following TBI that is at least in part mediated by NGF.

## Methods

### Animal care and housing

All experiments were performed in accordance with the National Institutes of Health Guide for Care and Use of Laboratory Animals. All experimental procedures were approved by Children's Memorial Research Center Institutional Animal Care and Use Committee. Adult male CD-1 mice weighing between 20-30 grams were used for these experiments.

### Mouse model of closed skull injury

Mice were subjected to closed skull injury using a stereotactically guided electromagnetic impactor (MyNeurolab, St. Louis MO) with minor modifications of published methods [26] as we have previously described [11]. Mice were anesthetized with isoflurane (4% induction, 1.5% maintenance) in 100% oxygen. Endotracheal intubation was performed and an 18 gauge angiocatheter was inserted as an endotracheal tube. Mice were mechanically ventilated (Hugo Sachs Electronik, March-Hugstetten, Germany), using a protective ventilation strategy (3 cm H_2_O PEEP; tidal volume 5 cc/kg) as previously described [27]. Core temperature was monitored using a rectal probe (IT-18 Physitemp, NJ) and maintained at 37.0 ± 0.1°C by surface heating and cooling. Mice were secured in prone position in a customized resin mold, and the scalp was shaved and prepared with betadine. A midline sagittal scalp incision was made using sterile technique, and the periosteum reflected to reveal the appropriate landmarks. A concave 3 mm metallic disk was affixed in the midline, immediately caudal to bregma. The zero depth position was determined by aligning the tip of the impact device in the down position with the surface of the skull. Closed skull injury was triggered using a Matlab-based computer controller (MyNeuroLab.com). A single controlled midline skull impact was delivered using a 2.0 mm steel tip impounder at a controlled velocity (3.0 ± 0.1 m/sec) and impact depth (3.0 mm). Mice with depressed skull fracture or visible hemorrhage were excluded from the study. After impact, the scalp incision was sutured and mice were allowed to achieve spontaneous respiratory effort prior to extubation.

### Histological preparation

Animals were sacrificed 1, 8, 14 or 28 days following TBI or sham procedure (n = 5 TBI and n = 5 sham animals at each time point). A subset of animals (n = 4) naïve to any type of surgical procedure including isofluorane anesthesia was also used as a separate control group. These latter animals were sacrificed 3 days after arrival to the animal facility. Mice were deeply anesthetized with a solution of tert-amyl alcohol and 2,2,2-tribromo ethanol (0.25 ml/10 grams body weight; Sigma, St. Louis, MO). After exposure of the chest cavity, animals were perfused via the left ventricle with 15 ml of heparinized phosphate-buffered saline (PBS), followed by a second 15 ml perfusion with 4% paraformaldehyde in PBS. Brains were dissected from the calvarium, immersed in 4% paraformaldehyde overnight at 4°C and subsequently transferred to 20% sucrose in PBS for cryoprotection. Brains were cut on a freezing microtome at a thickness of 40 μm and collected in PBS. The entire mouse brain was sectioned and the corpus callosum was present within approximately 72 sections. Every 12^th ^section was used for semi-quantitative analysis, yielding 5-7 sections per brain.

### Immunohistochemistry (IHC)

Immunohistochemical analysis was performed on free-floating 40-μm-thick sections. Resting and activated microglia were identified using an antibody to IBA-1 (1:1000; Wako, Richmond, VA). Activated microglia were identified using an antibody to galectin-3/Mac-2 (1:1000; R and D Systems, Inc., Minneapolis, MN). Control sections were incubated in normal serum or PBS in place of primary antibody.

Sections were rinsed in 0.01 M PBS and incubated in 1% hydrogen peroxide in PBS to quench endogenous peroxidase activity. Sections were rinsed again in PBS followed by incubation in 10% blocking serum. Normal goat serum (Vector Laboratories, Burlingame, CA) was used for ionized calcium binding adaptor molecule 1 (IBA-1)-IHC and normal rabbit serum (Vector) was used for galectin-3/Mac-2-IHC. Sections were incubated with primary antibody or control solution made in the same blocking serum with addition of 0.1% Triton-X for 48 hours at 4°C. Following incubation in primary antibody, sections were rinsed well in PBS and incubated in biotinylated secondary antibody (1:200; Vector) made in 1% blocking serum for one hour at room temperature. Sections were rinsed in PBS prior to incubating in Vectastain ABC solution, prepared per manufacturer's instructions. After further PBS rinses, the reaction product was visualized using diaminobenzidine substrate (DAB) as substrate (Vector). Sections were rinsed in PBS, mounted on slides and dried overnight. Slides were dehydrated in graded ethanol solutions, followed by xylene and coverslipped using DPX mounting medium.

### Immunofluorescence

White matter injury was assessed using an antibody to amyloid precursor protein (APP) (1:20000; Sigma, St. Louis, MO). Sections were rinsed well in PBS and incubated in 1% hydrogen peroxide. They underwent further PBS rinses before incubation in 10% blocking serum (normal goat serum, Vector). Following incubation in primary antibody overnight at room temperature, sections were rinsed well in PBS and incubated in secondary antibody (FITC conjugated chicken anti-rabbit (1:100; ABCAM; Cambridge, MA) made in 1% blocking serum for one hour at room temperature. Sections were rinsed well in PBS before mounting on slides and coverslipping with Fluorogel (Electron Microscopy Sciences, Fort Washington, PA). Slides were stored in the dark.

Double-labeling immunofluorescence studies were performed to identify which glial cell types were immunoreactive for NGF protein. Sections were rinsed well in PBS and incubated in 1% hydrogen peroxide. They underwent further PBS rinses before incubation in 10% blocking serum (made in normal swine serum (Vector)). Sections were then placed in the first primary antibody (anti-galectin-3/Mac-2 (1:500) or anti-IBA-1 (1:500) or rabbit anti-glial fibrillary acidic protein (GFAP)(1:2000; Dako, Carpinteria, CA) or rabbit anti-2',3'- cyclic nucleotide 3'-phosphodiesterase (CNPase) (1:1000; Sigma)) for 48 hours at 4°C or overnight at room temperature. After rinsing in PBS, sections were incubated in the dark in a solution containing the respective fluorescent secondary antibodies in 1% blocking serum. The following secondary antibodies were used: FITC conjugated chicken anti-rabbit (1:100; ABCAM) and donkey anti-goat Alexa Fluor 594 (1:100; Invitrogen Corporation, Carlsbad, CA). Following multiple rinses in PBS, sections were again placed in 10% blocking serum (normal swine serum) for one hour and then incubated in either rabbit anti-NGF (1:200; Sigma) or goat anti-NGF (1:1000; Abcam) either for 48 hours at 4°C or overnight at room temperature. Sections were rinsed well in PBS and incubated in the appropriate fluorescent secondary antibody as described above. Sections were rinsed well before mounting on slides and coverslipping with Fluorogel (Electron Microscopy Sciences, Fort Washington, PA).

**Figure 1:**
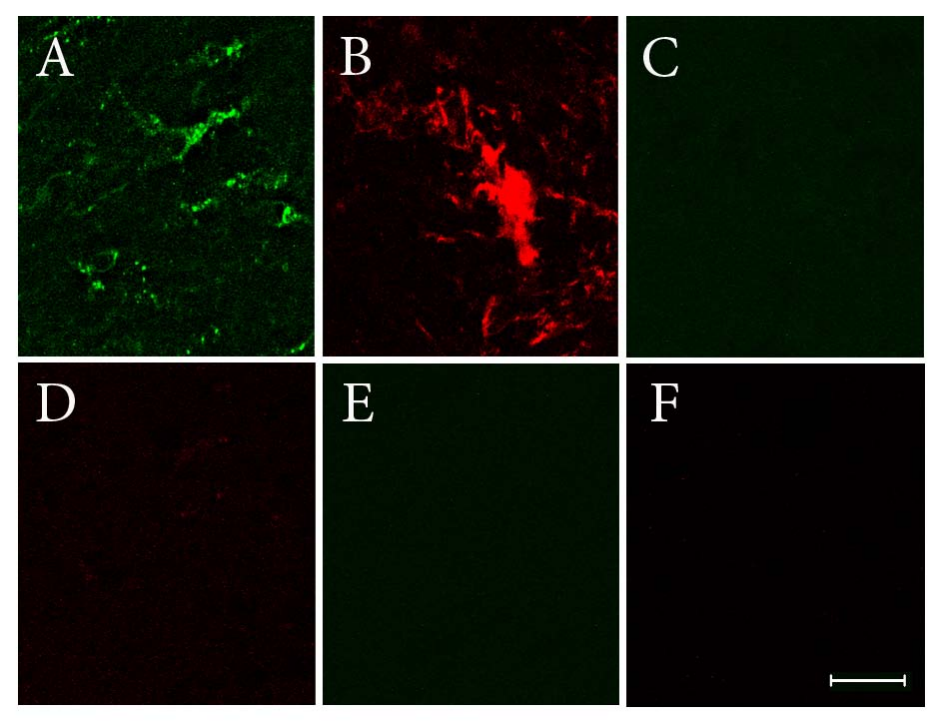
**Representative images of sections from control experiments of immunofluorescence labelling**. Panels A and B show immunoreactivity within the white matter for NGF (A) and galectin-3/Mac-2 (B). In panels C and D, sections were exposed to either goat anti-galectin-3/Mac-2 antibody (C) or rabbit anti-NGF antibody (D), but were then subsequently exposed to the inappropriate secondary antibody (FITC chicken anti-rabbit for galectin-3/Mac-2 (C) or Alexa Fluor 594 donkey anti-goat for NGF (D)). Panels E and F: Primary antibody was omitted and sections were exposed to either FITC chicken anti-rabbit (E) or Alexa Fluor 594 donkey anti-goat (F). These experiments verified that secondary antibodies did not bind to an inappropriate antigen in tissue sections. All tissues were from post-TBI (day 8) animals. Bar = 20 μm.

Control experiments for immunofluorescence studies were carried out with omission of one or the other primary antibodies and exposure to secondary antibodies. Experiments were also performed where a given primary antibody (e.g. goat anti- galectin-3/Mac-2) was exposed to the inappropriate secondary antibody (e.g. chicken anti-rabbit). These experiments verified that the secondary antibody had no target antigen in the tissue and thus provided confirmation that an inappropriate fluorescent signal was not produced (Figure 1).

### Electron microscopy processing

Animals were sacrificed after 24 hours recovery from TBI or sham procedure. All surgical procedures were identical to those used for light microscopy studies with the exception that a pneumatic impactor was used [11]. Qualitative analysis of APP-ir and galectin-3/Mac-2-ir via light microscopy confirmed no differences in the nature of injury using the pneumatic vs. electromagnetic impactor (data not shown). Animals underwent cardiac perfusion with 15 ml of heparinized PBS followed by 15 ml of 4% paraformaldehyde and 1% glutaraldehyde in PBS. Brains were removed from the calvarium and left in the fixative overnight at 4°C. Brains were then transferred to PBS and cut on a vibratome at a thickness of 40 μm. Sections were processed for galectin-3/Mac-2-IHC using DAB as the chromogen as described above (immunohistochemistry) with the exception that Triton-X was omitted from the incubation solution. Following completion of IHC, sections were rinsed in 0.1 M phosphate buffer (PB) and incubated in 1% osmium tetroxide for one hour and rinsed in 0.1 M PB. Sections were sequentially dehydrated in graded ethanol and acetone. They were incubated overnight in a mixture of Epon-812 (EMS, Hatfield, PA) and acetone. Sections were transferred to 100% Epon-812. Following incubation in resin for four hours, they were flat-embedded between sheets of Aclar plastic (EMS, Hatfield, PA) and baked in the oven at 80°C overnight. The region of interest within the corpus callosum was identified, cut out and embedded in BEEM capsules (EMS, Hatfield, PA). BEEM capsules were filled with resin and baked for 2 days at 80°C. Ultrathin sections were cut on a Leica ultramicrotome. Sections were counterstained with uranyl acetate and lead citrate and examined on a JEOL 1200 EX electron microscope. Images were digitally acquired on a Hamamatsu digital camera.

### Semi-quantitative analysis of galectin-3/Mac-2 and IBA-1- immunoreactive cells

Semi-quantitative analysis of galectin-3/Mac-2 and IBA-1-ir microglia was performed on every 12^th ^section (approximately 6 sections/animal). A Leica DMR-HC upright research microscope with an attached QImaging Retiga 4000R camera was used to obtain data images of the corpus callosum using a 20 × objective. Three non-overlapping adjacent montages encompassing the midline of the corpus callosum were photographed and stored for further analysis. Labelled cells were counted and areal measurements were made using Image J software. Only microglia with clearly visible cell bodies were counted.

### Qualitative and semi-quantitative analysis immunofluorescence labelling

Qualitative analysis of APP-ir was performed using a Zeiss 510 META confocal laser scanning microscope at low and high magnifications. Qualitative and semi-quantitative examination of double labelled fluorescent cells was performed on the confocal microscope at 63 × magnification using an oil immersion lens. For each animal, every 12^th ^serial section encompassing the corpus callosum was analyzed, yielding approximately 6 sections/animal. For semi-quantitative analysis of galectin-3/Mac-2 and NGF labelled cells, photographs of three non-overlapping montages of adjacent fields centered on the corpus callosum were taken with 63X oil immersion objective and stored for off-line analyses. For each field, photographs were taken of each data channel (e.g. galectin-3/Mac-2-ir and NGF-ir) separately as well as of the overlay image. Images were imported into Image J software. Single-labelled galectin-3/Mac-2-ir cells and double-labelled (galectin-3/Mac-2 and NGF-ir) cells were scored using different markers. A total of 196 cells were evaluated. Orthogonal reconstructions were also performed with a 63X oil immersion objective to view labelled cells in x-y, x-z and y-z cross-sectional planes to confirm that cells were double labelled (z-step, 1 μm). Values were exported to Excel spreadsheet and the percentage of double-labelled cells was calculated for each experimental group.

### Statistical analyses

Data were pooled within animals (across montages and tissue sections) and averaged across animals within a given experimental group. Values are expressed as mean ± SEM for each group. Two groups were compared using Student's t-test. Significance was defined as p < 0.05. Prism 4.0 (GraphPad Software, Inc., San Diego, CA) was used for statistical analyses.

## Results

### Closed skull impact produces axonal injury within the corpus callosum

**Figure 2:**
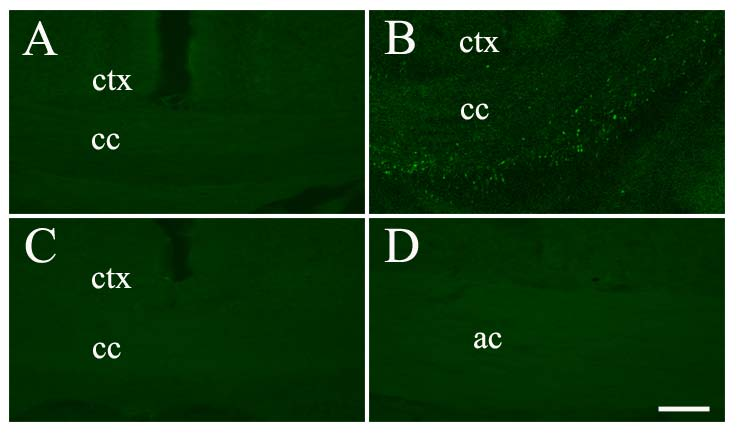
**Amyloid precursor protein (APP) immunoreactivity in sham and TBI animals**. APP-ir profiles are not evident within the corpus callosum (cc) in sham animals (A). One day post-TBI, prominent puncate labelling is present, reflecting accumulation of APP protein within damaged axonal endbulbs (B). This punctate pattern is not present 8 days post-TBI here (C) or in ventrally located white matter tracts such as the anterior commissure (ac) (D). ctx = cortex. Bar = 100 μm.

Mice were subjected to a closed-skull cortical impact or sham procedure. A group of mice naïve to any type of surgical intervention was also used. APP-ir has been used to evaluate axonal damage following TBI by others [28-32]. In our study, immunofluorescence staining for APP showed that sham animals did not have any labelled profiles within subcortical white matter tracts (Figure 2). In contrast, a punctate pattern of APP-ir, indicative of axonal injury, was apparent within dorsal midline white matter tracts such as the corpus callosum (Figure 2) and the cingulum at one day following TBI. At this time point, however, damaged axons were not noted within more laterally and ventrally situated subcortical white matter fibers including the lateral white matter tract and the anterior commissure (Figure 2). This punctate pattern was not evident by 8 days post-injury (Figure 2).

**Figure 3:**
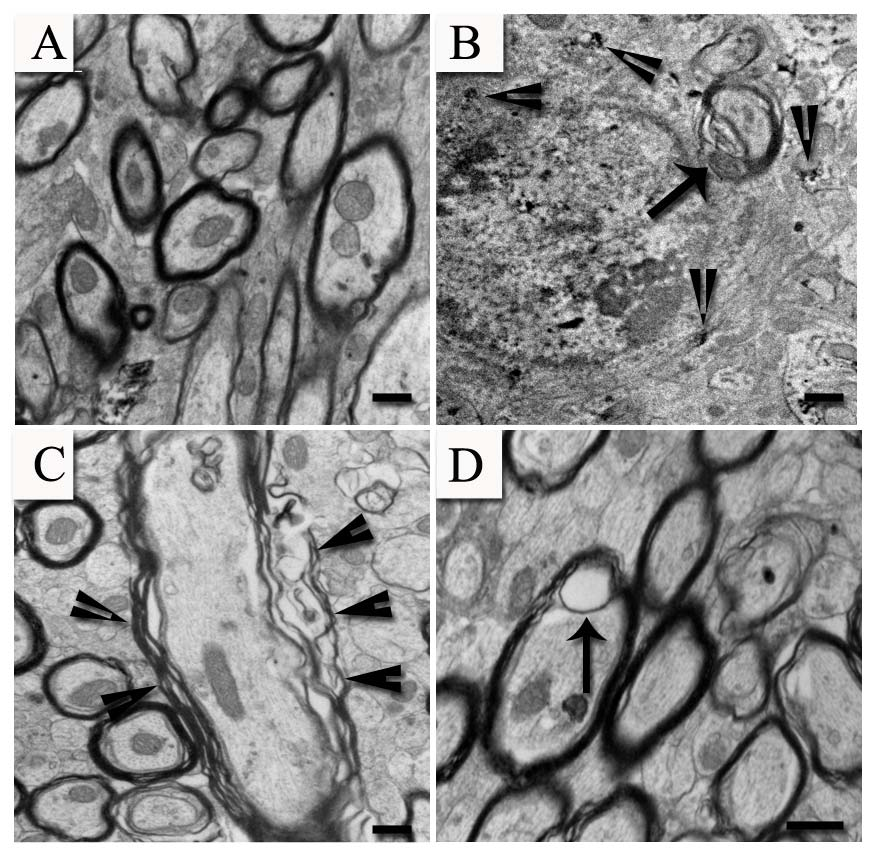
**Ultrastructural view of the corpus callosum in sham and TBI animals**. In sham animals, numerous intact myelinated axons with preserved cytoskeletal detail can be seen (A). In contrast, pathological changes are present in TBI animals (B, C, and D). Galectin-3/Mac-2+ microglial cell identified by the presence of DAB reaction product (arrowheads) is seen engulfing an injured axon (arrow in B). An example of an injured axon characterized by separation of the myelin sheath from the axolemma is shown (arrowheads in C). Focal swelling can also be seen within a damaged axon (arrow in D). Bar = 500 nm in A, 2 μm in B, 500 nm in C and D.

We used electron microscopy to evaluate ultrastructural features of damaged axons (Figure 3). Consistent with the changes in APP-ir observed at 24 hr following TBI, electron microscopic evaluation of the corpus callosum at the same time point showed pathological changes in the morphology of axons (Figure 3) as previously described following TBI [30,31,33-36]. In sham animals, ultrastructural examination of the corpus callosum showed intact myelinated axons with preserved cytoskeletal detail (Figure 3A). In contrast, numerous pathological changes were observed within the corpus callosum in TBI animals (Figure 3B, C and 3D). Following injury, there was separation of the myelin sheath from the axolemma. Intracellular blebs or focal edematous regions within axonal profiles were also observed. Within 24 hours following TBI, galectin-3/Mac-2-ir microglia within the corpus callosum were noted to be engulfing damaged axons (Figure 3B).

### Increase in IBA-1 immunoreactive microglia within the white matter after TBI

**Figure 4:**
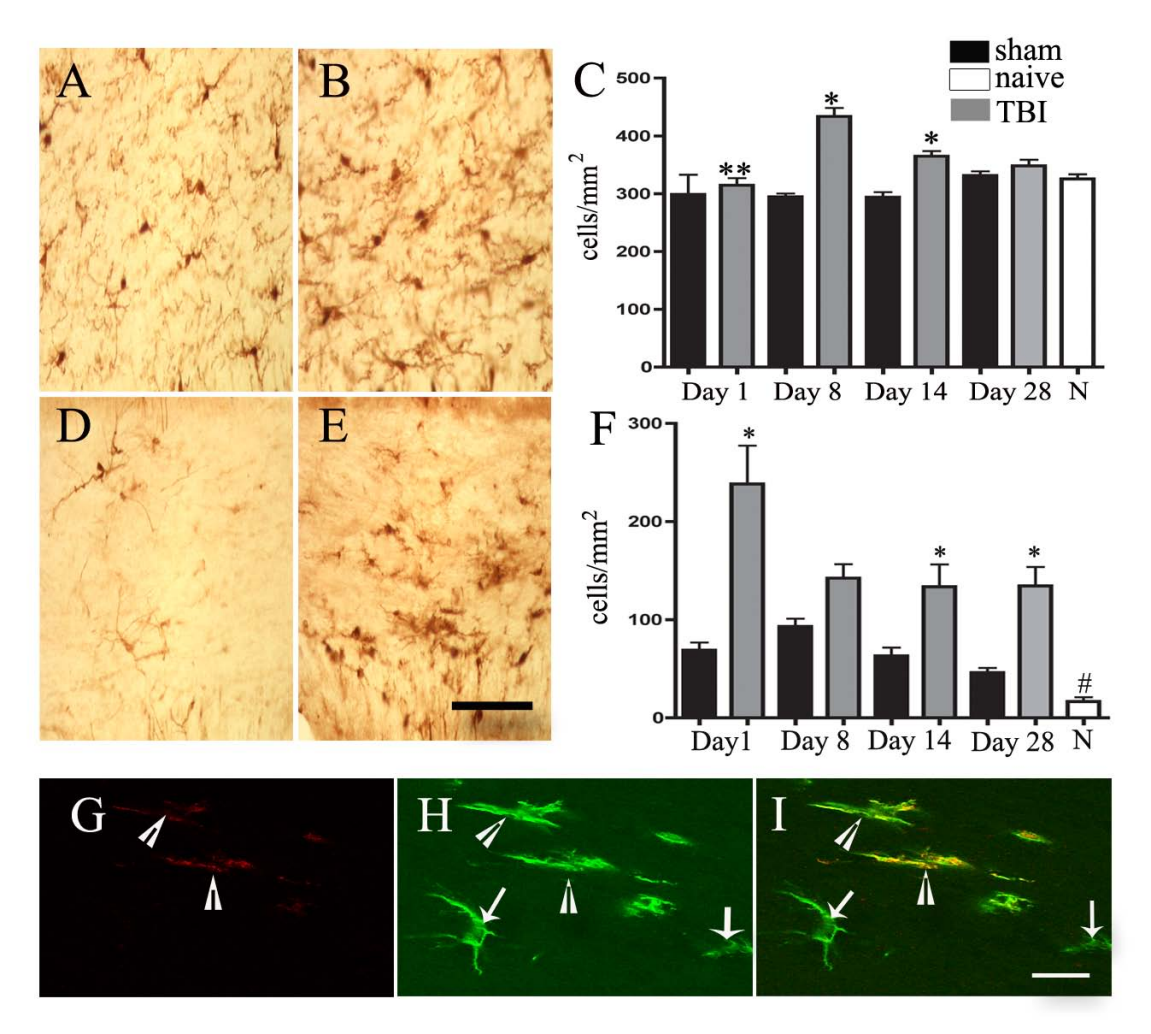
**IBA-1 and Mac-2 immunoreactivity following TBI**. Representative photomicrographs showing IBA-1-ir microglia scattered throughout the white matter in sham (A) and 8-day post-TBI (B) animals. Semi-quantitative analysis shows statistically significant increases in the density of IBA-1-ir microglia 1, 8 and 14 days following injury (C). *,p < 0.0001; **, p < 0.006 vs. sham. Representative photomicrographs showing pattern of galectin-3/Mac-2 labelling in uninjured (D) and injured 8 days post-TBI (E) animals. Semi-quantitative evaluation of the density of labeled cells confirms that following TBI, there is a significant increase in labelling (F) (*, p < 0.0001 vs. sham). There is also a significant difference in the density of labelling between naïve and sham animals at all time points (#, p < 0.0002 vs. sham). Bar = 100 μm in A-E. Representative photomicrographs illustrating microglia labeled for galectin-3/Mac-2 (G) or IBA-1 (H), and a pattern of co-localization (I). All galectin-3/Mac-2-labeled cells are immunoreactive for IBA-1, confirming that galectin-3/Mac-2-ir cells are microglia (arrowhead in panel G). However, there are IBA-1-ir cells that are not immunoreactive for galectin-3/Mac-2 (arrows in panels H and I), suggesting that IBA-1 labels a broader population of microglia. Bar = 20 μm in G-I.

In order to determine whether axonal injury identified in the corpus callosum was accompanied by changes in microglia subpopulations, we used immunohistochemical methods to detect alterations in IBA-1 immunoreactivity (Figure 4). IBA-1 protein is expressed within both active and resting microglia [16]. IBA-1-ir microglia were scattered within the corpus callosum of both injured and uninjured animals, but the density of IBA+ microglia was higher following injury (Figure 4). Semi-quantitative analysis revealed that following TBI, there was an increase in the density of IBA-1-ir microglia at all time points examined, reaching statistical significance on post-injury days 1, 8 and 14 (Figure 4; cell density (cells/mm^2^) ± SEM; naïve: 325.7 ± 7.843; sham day 1: 298.7 ± 34.12; TBI day 1: 314.6 ± 12.26 (p < 0.0006 vs sham day 1);; sham day 8: 294.8 ± 5.429; TBI day 8: 433.7 ± 14.76 (p < 0.0001 vs sham day 8); sham day 14: 293.9 ± 8.873; TBI day 14: 364.9 ± 8.896 (p < 0.0001 vs sham day 14); sham day 28: 331.3 ± 7.554; TBI day 28: 348.2 ± 10.53 (p < 0.225 vs sham day 28)).

### Chronic upregulation of galectin-3/Mac-2-ir microglia within the white matter after TBI

Galectin-3/Mac-2 immunoreactivity has been shown to be specific to a subset of activated microglia involved in myelin phagocytosis [20,22,23]. To examine the effects of TBI on this microglial subtype within the injured white matter, we evaluated the pattern of galectin-3/Mac-2 labelling in the corpus callosum. First, we confirmed by double labelling studies using anti-IBA-1 and anti-galectin-3/Mac-2 antibodies that all galectin-3/Mac-2+ cells were of microglial origin (Figure 4). Only a subpopulation of IBA-1-ir cells expressed galectin-3/Mac-2.

**Figure 5:**
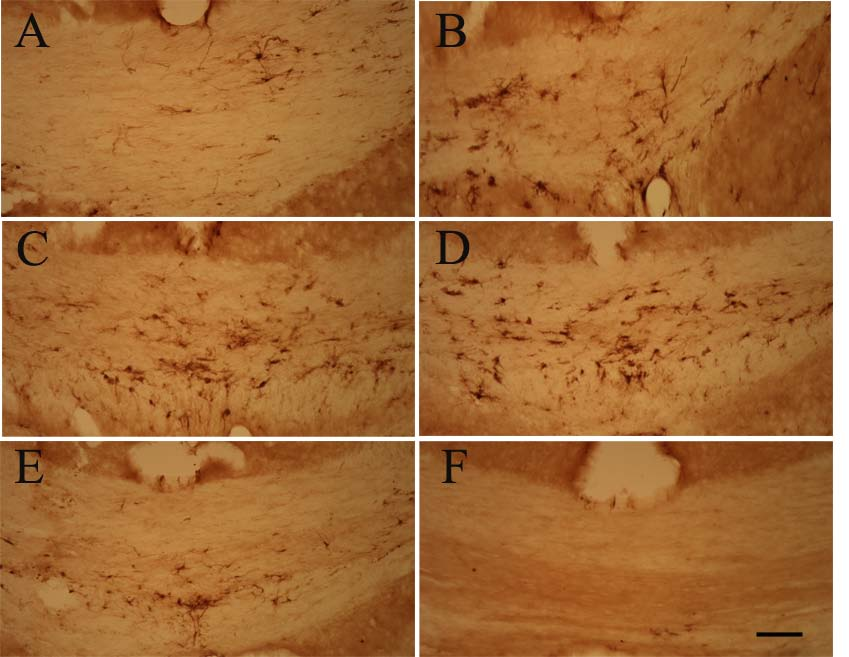
**Spatial pattern of galectin-3/Mac-2 immunoreactive cells within the corpus callosum following TBI**. Representative photomicrographs illustrating galectin-3/Mac-2-ir cells in the white matter in a TBI animal 24 after injury. Labelling within every 12^th ^serial section is shown. Qualitative assessment shows that the density of galectin-3/Mac-2 labelling is highest at the impact site (panels C and D). Labelling density decreases at levels both anteriorly (panels A and B) and posteriorly (panels E and F) from this site. Bar = 100 μm.

Qualitative analysis clearly revealed the presence of galectin-3/Mac-2-ir cells within the injured corpus callosum that varied in density most obviously in the anterior-posterior domain. The density of galectin-3/Mac-2+ microglia within the callosum was maximal beneath the site of impact and decreased both anteriorly and posteriorly from that site (Figure 5).

**Figure 6:**
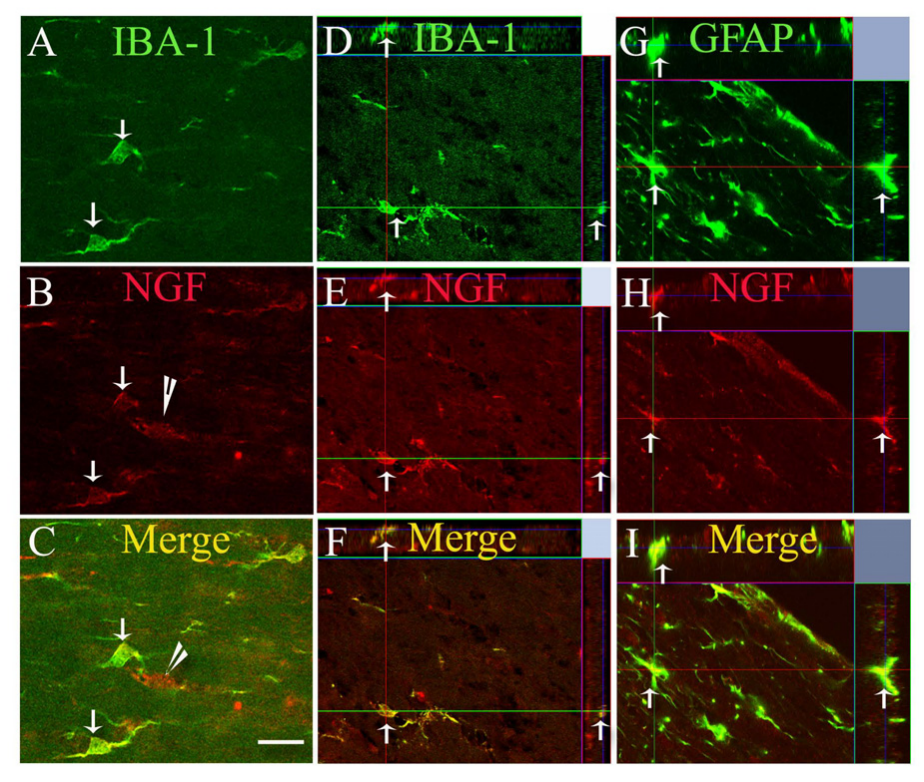
**Colocalization of NGF-ir cells with IBA-1-ir microglia and GFAP-ir glia**. Panels A-C show representative photomicrographs illustrating co-localization of IBA-1-ir (A) and NGF-ir (B) cells. Image overlay of NGF and IBA-1 labelling is shown in panel C. While the majority of cells are immunoreactive for both IBA-1 and NGF (arrows in A, B, and C), NGF-ir cells that are not IBA-1+ are also present (arrowhead in B and C). Bar = 20 μm in A-C. Orthogonal reconstructions from confocal z-series in x-z (top) and y-z (right) planes confirm that there is colocalization of IBA-1-ir and NGF-ir (D-F, arrows). Similar orthogonal reconstructions show colocalization of GFAP-ir and NGF-ir cells (G-I, arrows).

Semi-quantitative evaluation showed that there was a significant increase in galectin-3/Mac-2-ir microglia within the injured corpus callosum that was sustained 28 days post-TBI compared to sham animals (Figure 6; cell density (cells/mm^2^) ± SEM; naïve: 16.74 ± 4.253; sham day 1: 69.11 ± 7.706; TBI day 1: 238.3 ± 38.98 90 (p < 0.0001 vs sham day 1); sham day 8: 93.13 ± 8.045; TBI day 8: 142.3 ± 14.24 (p < 0.05 vs sham day 8); sham day 14: 63.25 ± 8.516; TBI day 14: 133.5 ± 22.90 (p < 0.0001 vs sham day 14); sham day 28: 46.04 ± 4.849; TBI day 28: 134.5 ± 19.24 90 (p < 0.0001 vs sham day 28)). The increase was most pronounced 24 hours following injury and was attenuated at later time points following injury (Figure 4). Interestingly, changes in galectin-3/Mac-2-ir microglia were also observed in sham animals. These animals were intubated and exposed to isofluorane anesthesia for the same duration of time as TBI animals; however, they only underwent skin incision, skin closure with sutures and were allowed to recover. Sham animals had a significant increase in galectin-3/Mac-2 labelling compared to naïve animals that were not exposed to any type of surgical procedure (Figure 4). This increase was at its peak 8 days post-injury and declined thereafter.

### Galectin-3/Mac-2-ir microglia are also immunoreactive for nerve growth factor protein

**Figure 7:**
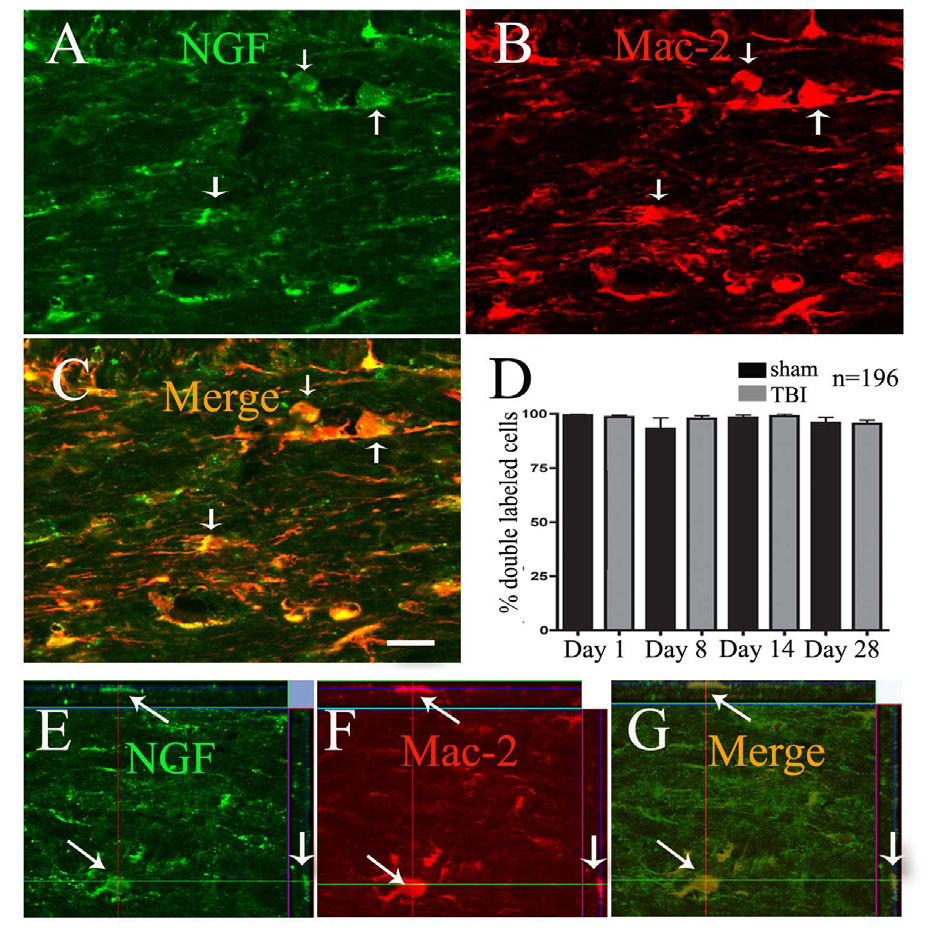
**Galectin-3/Mac-2 immunoreactive microglia are also immunoreactive for NGF**. Representative confocal photomicrographs illustrating co-localization of NGF-ir (A, arrows) and galectin-3/Mac-2-ir (B, arrows) microglia in an animal 7 days post-injury. Image overlay of NGF and galectin-3/Mac-2 labelling shows that the majority of galectin-3/Mac-2+ microglia are also immunoreactive for NGF. Semi-quantitative evaluation of percentage of double-labeled cells in all experimental groups confirms the qualitative observation; 93-99% of all galectin-3/Mac-2+ microglia are immunoreactive for NGF (D). Bar = 20 μm in A-C. Orthogonal reconstructions (E-G) from confocal z-series in x-z (top) and y-z (right) planes confirm that NGF-ir and galectin-3/Mac-2-ir are present within the same cell (arrows).

In order to investigate whether galectin-3/Mac-2-ir microglia were a source of the trophic factor NGF, we performed double labelling studies and visualized labeled cells using confocal microscopy. NGF-ir cells were found in the corpus callosum of both sham and TBI animals at all time points following injury. Qualitative evaluation of labelling clearly showed that the density of NGF-ir cells was higher following injury, paralleling the trend seen with galectin-3/Mac-2 labelling. Double-labelling immunofluorescence studies in tissue from both injured and uninjured animals revealed that the majority (93-99%) of galectin-3/Mac-2+ microglia were immunoreactive for NGF (sham day 1: 98.3 ± 1.2%; TBI day 1: 98.9 ± 1.0% 90 (p < 0.97 vs sham day 1); sham day 8: 99.4 ± 0.6%; TBI day 8: 98.5 ± 0.9% 90 (p < 0.6 vs sham day 8); sham day 14: 93.2 ± 5.0%; TBI day 14: 97.8 ± 1.3% 90 (p < 0.89 vs sham day 14); sham day 28: 96.0 ± 2.4%; TBI day 28: 95.5 ± 1.7% 90 (p < 0.46 vs sham day 28)) (Figure 7). However, NGF-ir cells that were not immunoreactive for galectin-3/Mac-2 were also present.

In order to determine whether NGF protein was present in other microglial subtypes, dual labelling immunofluorescence studies were performed using the microglial marker IBA-1. These studies identified two populations of NGF+ cells: one population was also immunoreactive for IBA-1 and the other was only immunoreactive for NGF (Figure 6). We further explored whether other glial cell types were immunoreactive for NGF. Double-labelling studies using an anti-CNPase antibody, a marker for mature oligodendrocytes failed to show colocalization between NGF and CNPase, suggesting that mature oligodendrocytes were not a source of NGF protein in the corpus callosum in sham and injured animals. In contrast, there was colocalization of NGF labelling with cells expressing the astrocytic marker GFAP (Figure 6). Thus, in addition to galectin-3/Mac-2-ir microglia, GFAP-ir astrocytes are also a source of NGF in the corpus callosum of injured and uninjured mice.

## Discussion

This study, using a murine model of closed skull diffuse head injury, shows that there is chronic upregulation of a sub-population of microglia immunoreactive for galectin-3/Mac-2 within the corpus callosum. Ultrastructural evaluation of the callosum using electron microscopy and qualitative examination of APP-ir using light microscopy both demonstrate axonal injury following this insult. Axonal injury was confined to dorsal midline white matter tracts such as the corpus callosum and the cingulum. Injured axons were not noted within more laterally and ventrally situated subcortical white matter tracts such as the anterior commissure. This may relate to the physical proximity of the area of impact overlying the dorsal midline. The presence of galectin-3/Mac-2-ir microglia within the injured white matter in the setting of diffuse axonal injury is consistent with the previously identified role for these microglia in myelin phagocytosis following central nervous system inflammation [20,22,23]. We also show that this subpopulation of microglia is immunoreactive for nerve growth factor in naïve, sham and injured animals at all time points examined. Our findings suggest that galectin-3/Mac-2-ir microglia are involved in the pathogenesis of diffuse axonal injury both acutely and chronically and that they mediate their effects, at least in part by releasing NGF.

The increase in activated microglia identified by galectin-3/Mac-2 appears to vary with the type and severity of neurologic insult. Previous studies have reported the presence of galectin-3/Mac-2 expressing microglia at sites of neuronal damage following hypoxia-ischemia injury [20], but not after facial nerve axotomy [20]. The authors observed that these two models differ in the degree of severity of injury and concluded that galectin-3/Mac-2 expression correlates with microglial activation in response to severe trauma [20]. In the murine peripheral nervous system, sciatic nerve transection induces Schwann cells to express galectin-3/Mac-2 and phagocytose myelin [22].

A number of lines of evidence support a role for this population of microglia in the mechanisms of phagocytosis following cerebral injury. It has been proposed that Mac-2 mediates myelin phagocytosis in the periphery through a non-immune, opsonin-independent mechanism referred to as lectinophagocytosis [22]. In support of a role for galectin-3/Mac-2 in active myelin phagocytosis, it was shown in a model of optic nerve injury, where myelin degradation and phagocytosis proceed slowly, that only a small subpopulation of microglia express galectin-3/Mac-2 [14]. In contrast, after experimental allergic encephalomyelitis in mice, sites of demyelination within the spinal cord and optic nerve with active myelin degeneration and phagocytosis displayed robust expression of galectin-3/Mac-2 by resident microglia [23]. In vitro studies further support a role for galectin-3/Mac-2 expressing microglia in myelin phagocytosis [21,23,37]. In our studies, galectin-3/Mac-2-ir microglia are robustly upregulated within the corpus callosum following diffuse TBI, and we find ultrastructural evidence one day following injury that these microglia engulf damaged axons. This finding supports at least one specific role for galectin-3/Mac-2-ir microglia acutely following diffuse axonal injury, the phagocytosis of myelin in this injury model as well.

There is emerging evidence that galectin-3/Mac-2-expressing microglia have roles beyond myelin phagocytosis. Selective ablation of galectin-3/Mac-2-positive microglia in transgenic mice was associated with an increase in the size of the infarct zone following transient middle cerebral artery occlusion [16]. Further, galectin-3/Mac-2-ir microglia expressed insulin-like growth factor (IGF), suggesting that this subset of microglia has a neuroprotective role via secretion of trophic growth factors [16]. Long term upregulation of both IBA-1-ir and galectin-3/Mac-2-ir microglia has also been observed in the subventricular zone (SVZ) following middle cerebral artery occlusion [18]. IBA-1-ir microglia in these studies colocalize IGF, which supports a neuroprotective and supportive role for microglia in the generation of neuroblasts within the SVZ [18].

Our results identifying a subset of galectin-3/Mac-2-ir microglia also immunoreactive for the neurotrophic factor NGF, support a dual role. NGF has been shown to be elevated in cerebrospinal fluid of children following traumatic brain injury [24,25]. These studies noted a correlation between NGF level and outcome; better clinical outcome was correlated with higher NGF levels [24,25] suggesting that NGF may have a beneficial role in neuronal repair. Experimental studies of exogenous neurotrophin administration show beneficial effects on axon sprouting, neuronal survival and oligodendrocyte proliferation [38-43].

However, there is also evidence for a detrimental role for NGF and its precursor proNGF. NGF has been implicated in the etiology of autonomic instability present after spinal cord injury, which can be blocked by intrathecal administration of neutralizing antibody to NGF or trkA-IgG fusion protein that binds NGF [44,45]. NGF's growth-promoting effects are mediated via binding and activation of high affinity trkA Neurotrophin receptor [for review, see [46]]. In the absence of trkA, NGF binds to its low affinity receptor p75NTR, which is thought to be responsible for triggering death pathways [47]. Pro-NGF, which binds with high affinity to p75NTR, and NGF have been implicated in p75NTR-mediated oligodendrocyte death in the retina and spinal cord [48-51]. Our studies find both an acute increase in galectin-3/Mac-2+/NGF+ microglia 1 day following injury as well as a relatively attenuated chronic upregulation of this population of microglia even 28 days following injury. It is not clear whether the functional properties of this microglial subpopulation remain the same during the acute and chronic phases of injury. It is possible that the effector functions of NGF depend on the populations of mature oligodendrocyte and oligodendrocyte precursors present at various time frames following injury and the neurotrophin receptors found on these cells.

Our study also found a statistically significant increase in galectin-3/Mac-2-ir microglia within the corpus callosum in animals exposed to sham surgery when compared to naïve animals; however, this increase in labelling in sham animals was still significantly lower than that seen following TBI and declined over time. The reason for this increase is not readily apparent, but may involve a generalized stress response to the sham surgical procedure. Morphological, but not functional activation of microglia within the central nervous system has been noted in rodents following restraint stress and immersion of body in water [52].

## Conclusions

The results from this study, using an in vivo model of closed skull injury, provide further evidence that specific populations of microglia are differentially activated in response to brain. We show that following diffuse TBI, activated microglia expressing the lectin galectin-3/Mac-2 are chronically upregulated within the corpus callosum. Furthermore, the majority of these microglia are immunoreactive for nerve growth factor. Our results support the growing body of evidence that there are heterogenous subpopulations of microglia, which secrete different neural effectors including growth factors at varying points following injury. The elucidation of the mechanisms by which microglia are activated and contribute to mechanisms of injury or repair following TBI will be essential if activated microglia are to serve as a therapeutic target for reducing neurologic morbidity associated with white matter injury after head injury.

## Competing interests

The authors declare that they have no competing interests.

## Authors' contributions

CV contributed to the design of the study, analyzed the data, performed the statistical analysis and wrote the manuscript; MC performed animal surgeries, reviewed the data and the manuscript; NC performed immunofluorescence studies, reviewed the data and the manuscript; MSW contributed to the design of the study, provided consultation and critically revised the manuscript. All authors read and approved the final manuscript.
